# Prognostic significance of serum fucosylated pro-haptoglobin in advanced renal cell carcinoma patients treated with immune checkpoint inhibitors

**DOI:** 10.1038/s41598-023-42739-1

**Published:** 2023-10-11

**Authors:** Taigo Kato, Koichi Morishita, Eisuke Tomiyama, Ayumu Hayashibara, Yu Ishizuya, Yoshiyuki Yamamoto, Koji Hatano, Atsunari Kawashima, Shinichiro Fukuhara, Norio Nonomura, Eiji Miyoshi, Kazutoshi Fujita

**Affiliations:** 1 Department of Urology, Osaka University Graduate School of Medicine, 2-2 Yamadaoka, Suita, 565-0871 Japan; 2 Department of Molecular Biochemistry and Clinical Investigation, Osaka University Graduate School of Medicine, Suita, Japan; 3 Department of Urology, Kindai University Graduate School of Medicine, Sayama, Japan

**Keywords:** Cancer, Biomarkers, Medical research, Oncology, Urology

## Abstract

With the widespread use of immune checkpoint inhibitors (ICIs), identifying predictive biomarkers is critical. Recently, serum fucosylated haptoglobin (Fuc-Hp) was thought to play an important role in tumour immunity in several types of cancer. Therefore, evaluating serum Fuc-Hp in the peripheral blood can potentially identify non-invasive predictive biomarkers for the clinical efficacy of ICIs. In this study, 31 patients with advanced renal cell carcinoma (RCC) treated with nivolumab were enrolled and defined as responders or non-responders according to RECIST criteria. Serum samples were collected before and 1 month after treatment initiation, and an ELISA assay was performed using *Aleuria Aurantia Lectin* (AAL) and 10-7G monoclonal antibodies that recognise Fuc-mature Hp (Fuc-mHp) and Fuc-pro Hp (Fuc-pHp), respectively. We first measured AAL-haptoglobin (Fuc-mHp) and total haptoglobin levels before nivolumab and found that neither value could predict the clinical response. Notably, serum 10-7G levels were significantly lower in the responder group (*p* = 0.035). We also confirmed the use of serum 10-7G levels for predicting progressive disease after nivolumab (area under the curve, 0.816). Accordingly, low 10-7G levels were significantly correlated with better progression-free survival (*p* = 0.041). In conclusion, serum Fuc-pHp analysis may identify patients with advanced RCC who benefit from ICIs.

## Introduction

Recent progress in cancer immunotherapy has revolutionised immunotherapeutic approaches to cancer treatment and has remarkably improved the prognosis of cancer patients^1–3^. In particular, immune checkpoint inhibitors (ICIs) have provided a paradigm shift in therapy for several types of cancer^4^. However, response rates to ICIs remain limited, and most patients fail to respond to ICIs^5,6^. To date, programmed death-ligand 1 (PD-L1) expression in tumours or a high tumour mutation burden has been thought to demonstrate improved clinical outcomes after ICI treatment^6^. However, these markers mainly focus on the tumour state at the time of cancer diagnosis and sometimes show contradictory outcomes because tumours generally undergo drug-imposed selective pressure before the initiation of ICI treatment. Furthermore, diagnostic tumour biopsies immediately before ICI treatment are impractical for many cancers, except melanoma. To overcome these limitations, there is an urgent need to develop decisive and non-invasive biomarkers to stratify responders and improve the clinical efficacy of cancer precision medicine.

Fucosylation is one of the most important glycosylation reactions involved in the progression of various types of cancer^7^. In our previous studies, lectin-antibody enzyme-linked immunosorbent assay (ELISA) was used to measure serum fucosylated haptoglobin (Fut-Hp) levels and revealed significantly increased serum Fut-Hp levels in several types of cancer and inflammatory bowel diseases^8–10^. Recent studies have highlighted the importance of fucosylation in immune cell development and functional regulation and have significantly broadened the role of fucosylation in cancer immunity and inflammatory diseases^8,11,12^.

In the present study, we evaluated serum Fuc-mature Hp (Fuc-mHp) and Fuc-pro Hp (Fuc-pHp) levels in patients with metastatic renal cell carcinoma (mRCC) treated with nivolumab (anti-programmed death protein 1 [PD-1] antibody) using our developed antibodies^13^. Furthermore, we investigated whether Fuc-Hp levels can be a potential biomarker for predicting the clinical prognosis in mRCC patients with ICI treatment.

## Results

### Patients’ characteristics

Patients’ clinical characteristics patients are summarized in Table 1. Twenty-eight tumours (90.3%) were histologically diagnosed as clear cell RCC. The median age of the patients was 65 years (range 45–81 years). Twenty-two patients (78.6%) received nivolumab as a secondary treatment. All patients received at least one treatment for metastatic cancer before receiving nivolumab. The best overall responses were CR in 1 patient, PR in 5 patients, and stable disease (SD) in 17 patients, resulting in an objective response rate (ORR) of 19.4% and a clinical benefit rate of 74.2% (Table 1). In the present study, considering distinctive radiographic responses to nivolumab^1^, we defined responders as patients with CR, PR, or SD for ≥ 6 months (long SD [LSD]). Therefore, responders and non-responders comprised 21 and 10 patients, respectively.

**Table 1:** Patients’ characteristics.

Characteristics	
Median age, years (range)	65 (45–81)
Gender, n (%)	
Male	27 (87.1)
Female	4 (12.9)
Histopathology, n (%)	
Clear cell RCC	28 (90.3)
Papillary RCC	1 (3.2)
Unclassfied	1 (3.2)
Collecting duct carcinoma	1 (3.2)
IMDC risk, n (%)	
Favorable	2 (6.5)
Intermediate	24 (77.4)
Poor	5 (16.1)
Treatment lines of Nivolumab, n (%)	
2nd	22 (71.0)
≧3rd	9 (29.0)
Number of metastatic organs, n (%)	
Single	13 (41.9)
Multiple	18 (58.1)
Metastatic site	
Bone	7 (22.6)
Lung	15 (48.4)
Liver	4 (12.9)
Lymph nodes	10 (32.3)
Other	8 (25.8)
Best objective response, n (%)	
CR	1 (3.2)
PR	5 (16.1)
SD ≥ 6 M	15 (48.4)
SD < 6 M	2 (6.5)
PD	8 (25.8)
ORR	6 (19.4)
CBR	21 (74.2)

*CBR* clinical benefit rate, *CR* complete response, *ORR* objective response rate, *PD* progressive disease, *PR* partial response, *RCC* renal cell carcinoma, *SD* stable disease.

### The responders' serum 10-7G level at baseline was significantly lower

We previously developed lectin *Aleuria Aurantia Lectin* (AAL) and 10-7G monoclonal antibodies that mainly recognise Fuc-mHp and Fuc-pHp, respectively (see Supplementary Fig. S1 online)^13^.

**Figure 1:**
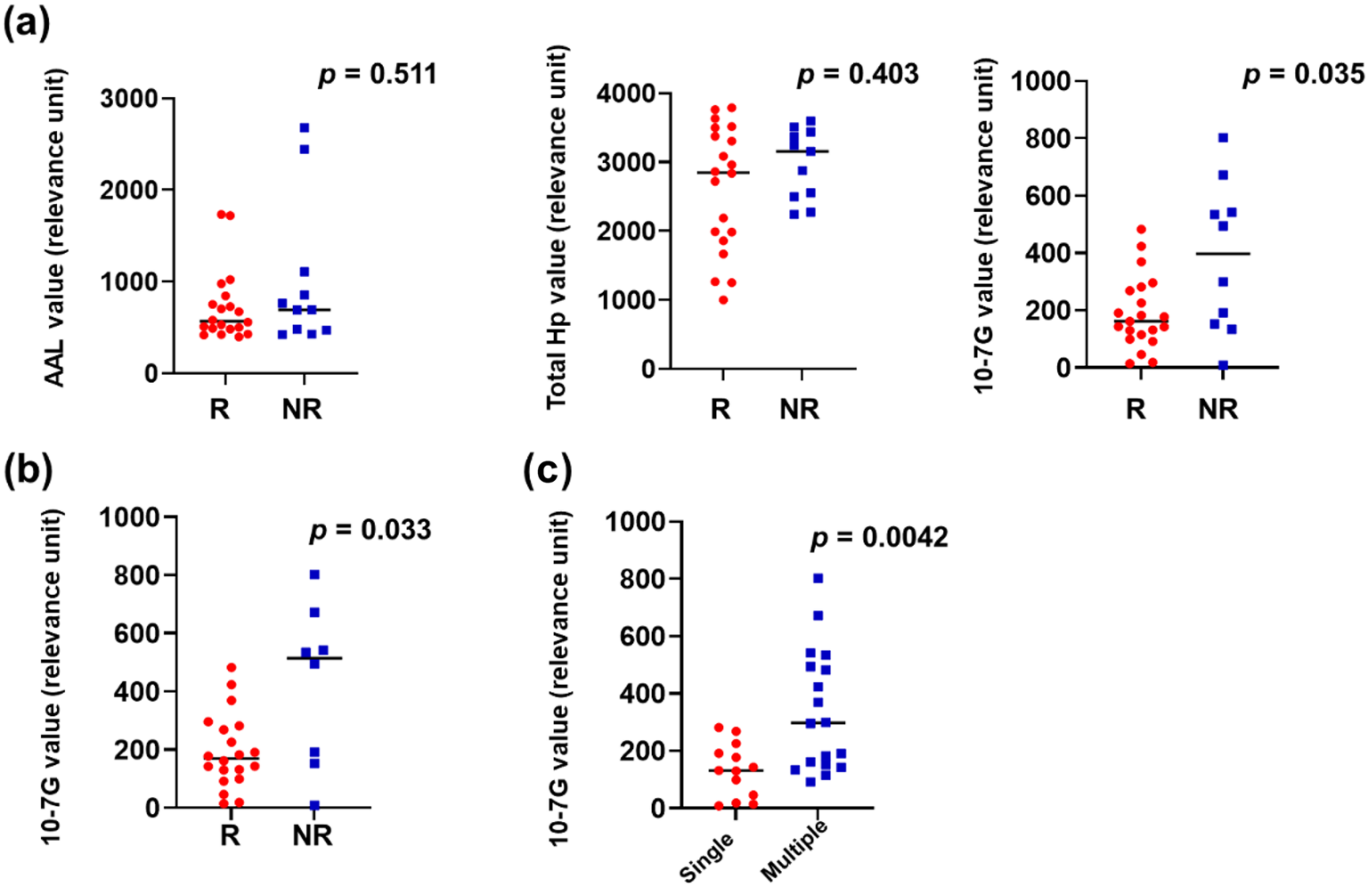
Low 10-7G level in responders treated with nivolumab monotherapy. (**a**) Responders showed significantly lower 10-7G level, not AAL and total haptoglobin levels in the peripheral blood before treatment initiation (versus non-responders). R, Responders; NR, Non-responders. (**b**) Responders had significantly lower 10-7G levels in the peripheral blood before treatment initiation (vs. non-responders), even when we focused on the patients with clear-cell renal cell carcinoma. R, Responders; NR, Non-responders. (**c**) Serum 10-7G level in cases of single and multiple metastatic sites.

Using these antibodies, we performed an ELISA test for serum obtained from patients with mRCC before the initiation of nivolumab therapy to examine the effects of Fuc-mHp and Fuc-pHp. We first measured the AAL (Fuc-mHp) and total Hp levels; notably, AAL and total Hp levels before the initiation of nivolumab therapy could not predict clinical response in the present study (*p* = 0.511 and *p* = 0.403, respectively; Fig. 1a).

We further investigated the serum 10-7G level (Fuc-pHp) and found that the 10-7G level before nivolumab therapy was significantly lower in responders (*p* = 0.035; Fig. 1a). We also found that the 10-7G level in responders was significantly lower when compared to that in non-responders in patients with clear-cell RCC (*p* = 0.033 Fig. 1b). Even when we focused on patients who received nivolumab as second-line treatment, the 10-7G level tended to be lower in the responders (*p* = 0.104; see Supplementary Fig. S2 online). Next, we examined whether the number of metastatic site was correlated with 10-7G level and found that patients with single metastasis had a significantly lower 10-7G level than those with multiple metastases (*p* = 0.0042; Fig. 1c).

**Figure 2:**
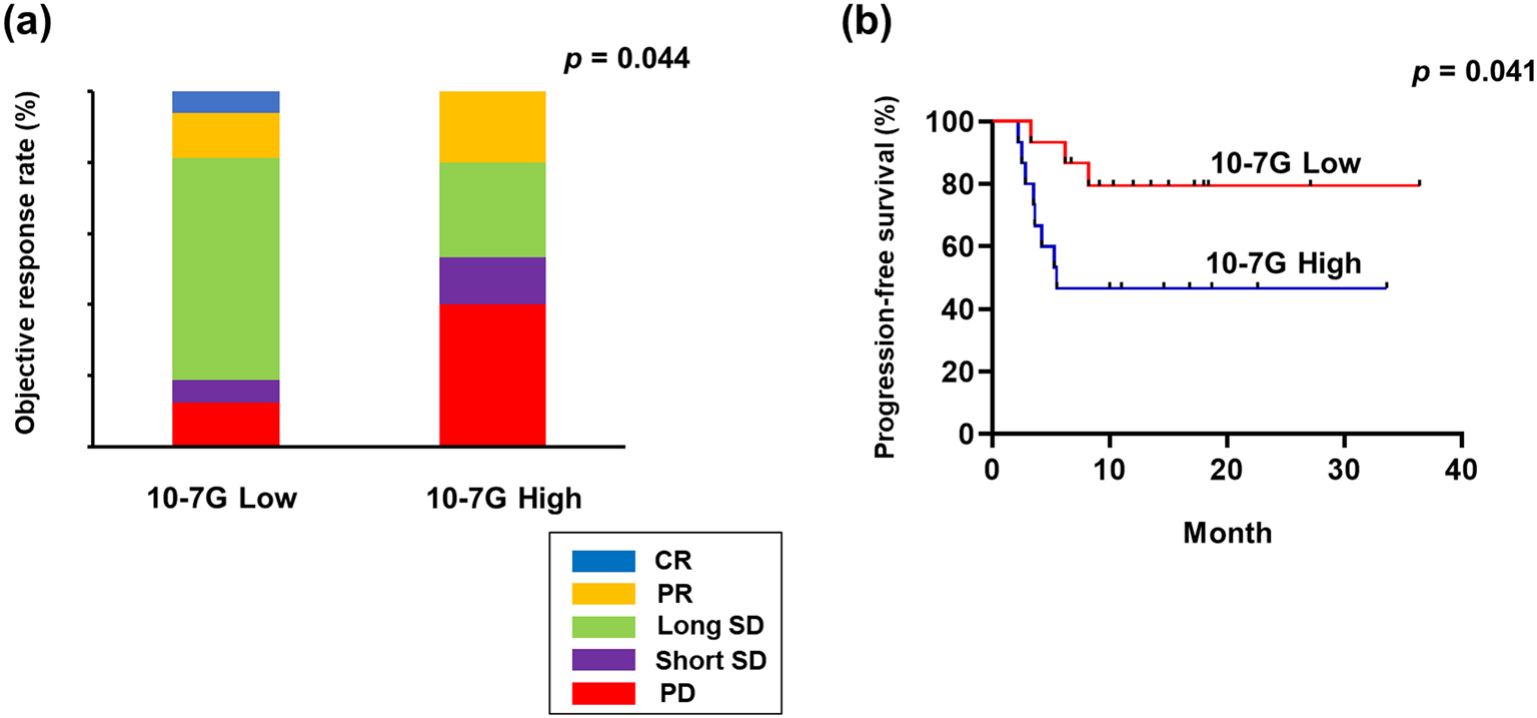
Clinical utility depending on 10-7G level in the peripheral blood of responders to nivolumab. (**a**) Objective response rate during nivolumab treatment according to serum 10-7G level before treatment initiation. *CR* complete response; *PR* partial response, *PD* progressive disease, *SD* stable disease. (**b**) Progression-free survival rate of patients treated with nivolumab. Survival rates were compared alternatively between patients with low 10-7G (n = 16) and high 10-7G (n = 15) levels.

Subsequently, we divided the patients into two groups based on the median serum 10-7G level (182.5, relevance unit). Notably, CR, PR, and LSD accounted for 81.3% of the patients with a low 10-7G level (low 10-7G group), whereas 53.3% of the patients with a high 10-7G level (high 10-7G group) had short SD (SSD) and PD (Fig. 2a). Accordingly, the progression-free survival (PFS) after the initiation of nivolumab therapy was significantly longer in the low 10-7G level group than in the high 10-7G level group (*p* = 0.041; Fig. 2b), whereas the median 10-7G level 1 month after treatment initiation did not influence the clinical outcome (see Supplementary Fig. S3 online).

These results imply that serum Fuc-pHp level at baseline is an informative and feasible therapeutic biomarker for patients undergoing anti-PD-1 therapy, compared with Fuc-mHp level at baseline.

### The fucosylated pro-haptoglobin level is dominant in patients with metastatic renal cell carcinoma

**Figure 3:**
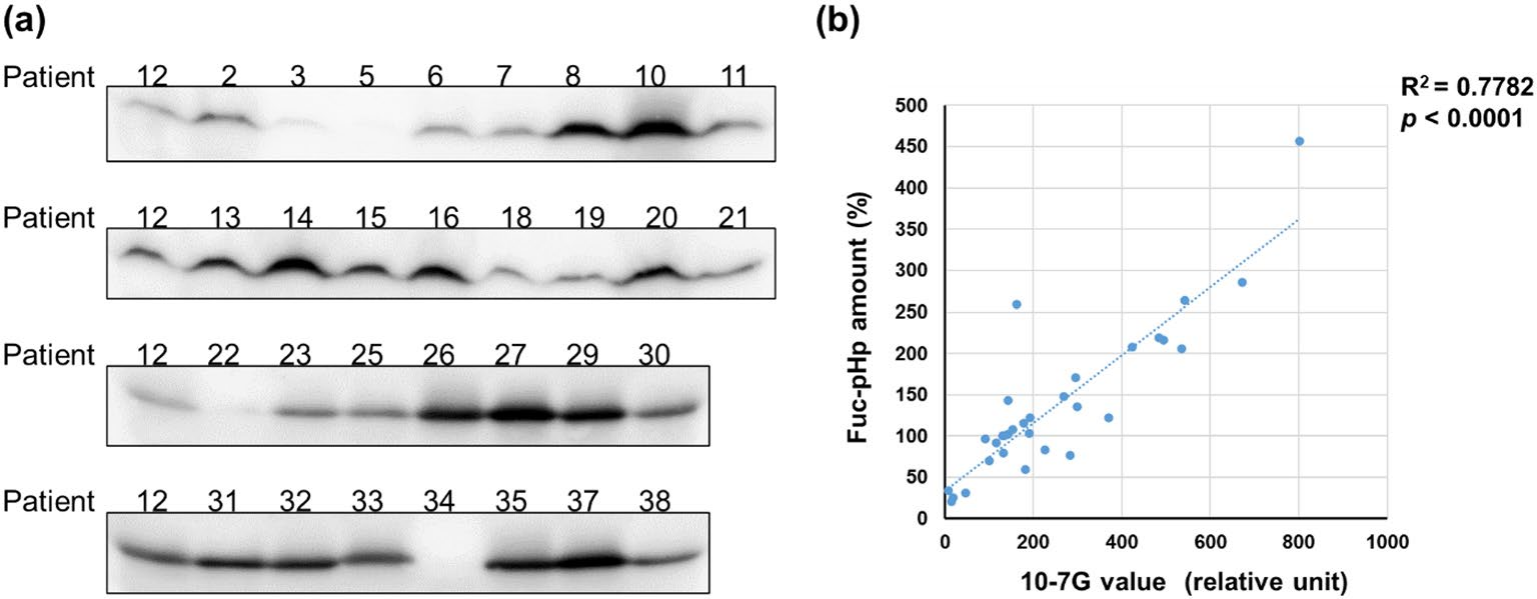
Measurement of the serum 10-7G and fucosylated pro-haptoglobin levels in patients with metastatic renal cell carcinoma treated with nivolumab. (**a**) Semi-quantification of serum fucosylated pro-haptoglobin (Fuc-pHp) level using immunoblot analysis using 10-7G antibody. Original data is shown in Supplementary Fig. S5 online. (**b**) Correlation between Fuc-pHp level using immunoblot analysis and 10-7G level using ELISA.

Next, we evaluated serum AAL Fuc-mHp and 10-7G Fuc-pHp levels, and no significant correlation was found between serum AAL and 10-7G levels at baseline (R^2^ = 0.061, *p* = 0.108; see Supplementary Fig. S4 online). Using immunoblot analysis, we further assessed serum Fuc-pHp levels using the 10-7G monoclonal antibody (mAb) described as the Fuc-pHp amount (Fig. 3a). Notably, as shown in Fig. 3b, a significant positive correlation was observed between the 10-7G and Fuc-pHp levels (R^2^ = 0.7782, *p* < 0.0001). These results indicate that cancer tissues in patients with mRCC mostly produce Fuc-pHp, not Fuc-mHp.

### Evaluation of 10-7G level for stratifying progressive disease after nivolumab therapy

**Figure 4:**
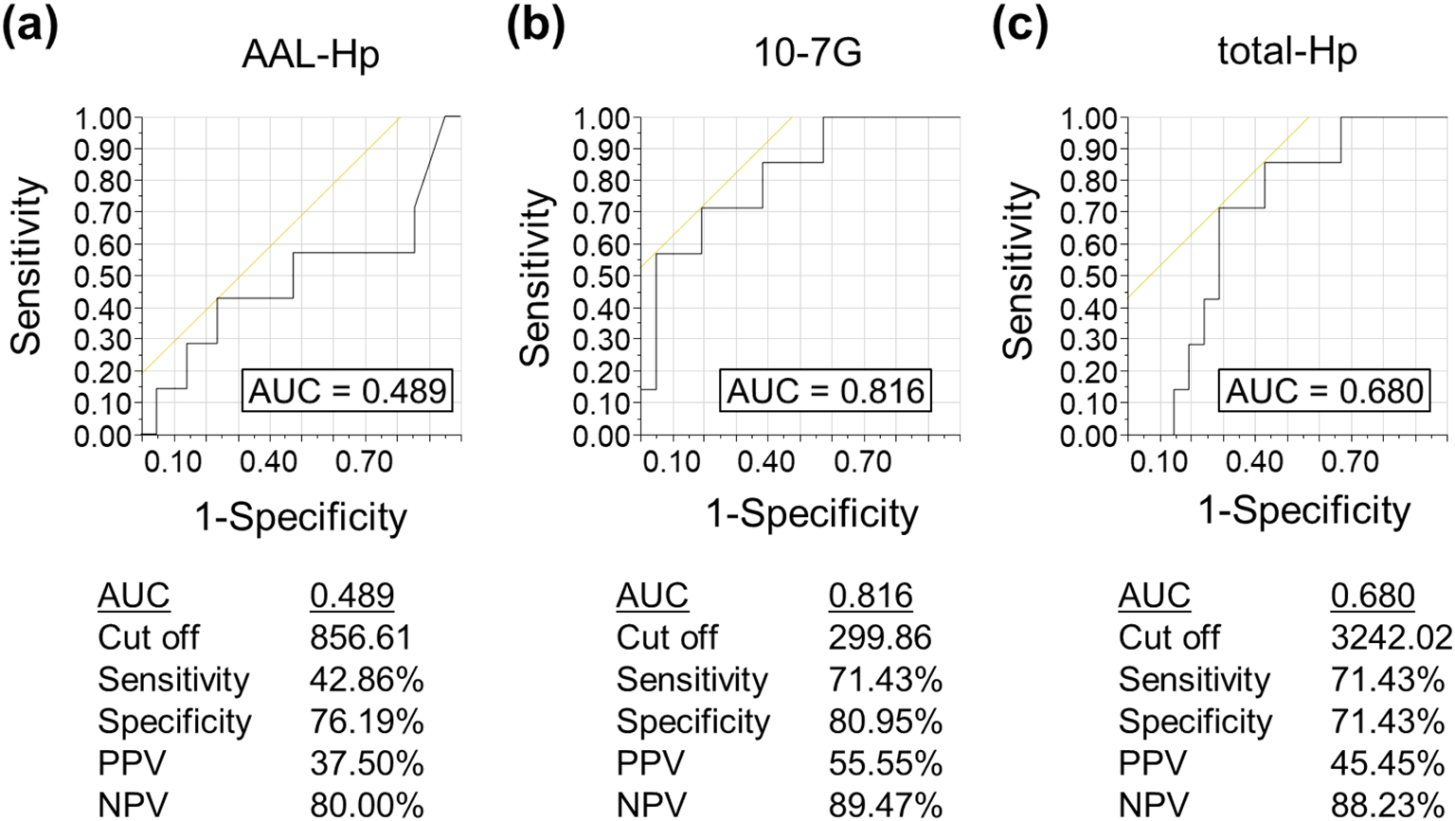
Receiver operating characteristic curves of the predicted probability of progressive disease according to AAL-haptoglobin, 10-7G, and total-haptoglobin levels in patients with metastatic renal cell carcinoma treated with nivolumab. (**a**–**c**): Analysis of receiver operating characteristic curves for AAL-haptoglobin (AAL-hp), 10-7G, and total haptoglobin (total-Hp) levels to differentiate between progressive disease patients with others. *AUC* Area under the curve.

The sensitivity and specificity of the serum 10-7G, AAL-Hp, and total Hp levels for predicting PD were determined using receiver operating characteristic curve analysis. We calculated the area under the curve (AUC) for the 10-7G level. Using the 10-7G level, the AUC for predicting PD was 0.816, whereas the AUC for AAL-Hp and total Hp levels were 0.489 and 0.680, respectively (Fig. 4).

Furthermore, univariate and multivariate analyses revealed that a low 10-7G level was significantly associated with better PFS (*p* = 0.048 and 0.044, respectively; Table 2).

**Table 2:** Univariate and multivariate analyses to predict long PFS.

Variable	PFS			
	Univariate analysis		Multivariate analysis	
	HR	*p* value	HR	*p* value
Age				
< 65	Reference			
≥ 65	2.104 (0.632–7.004)	0.225	2.354 (0.603–9.187)	0.218
Histology				
Clear	Reference			
Non-clear	3.449 (0.737–16.138)	0.169	2.822 (0.508–15.684)	0.236
IMDC risk				
Favorable	Reference			
Intermediate/poor	2.012 (0.201–7.660)	0.156	6.131 (0.535–3.968)	0.279
10-7G value				
Low	Reference			
High	3.394 (0.914–12.599)	0.048	4.895 (1.002–23.903)	0.044
Number of metastatic sites				
Single	Reference			
Multiple	2.694 (0.728–9.967)	0.112	1.101 (0.223–5.351)	0.914

*HR* hazard ratio, *IMDC* International Metastatic RCC Database Consortium.

These findings indicate that the 10-7G level is a useful and precise biomarker for predicting PD in patients with mRCC treated with nivolumab.

## Discussion

Immunotherapy targeting immune checkpoints is currently the most attractive therapy in the cancer field and has improved the prognosis of cancer patients^14–16^. However, the response rate to ICI monotherapy ranges from 11 to 40%, and treatment-related toxicity often causes discontinuation of ICIs therapy^17^. Therefore, it is crucial to identify biomarkers to predict the patients’ response and clinical prognosis to immunotherapeutic strategies to enable precision medicine. To date, studies on predictive markers of response have mainly focused on evaluating untreated tumour tissues before the initiation of drug therapies and have produced conflicting findings^18^.

Fucosylation is an oligosaccharide modification that plays a critical role in immune response and malignancy^12,19–21^. Recently, fucosylation was reported to be associated with different physiological and pathological processes depending on the tumour type, focusing on its roles in cancer cell proliferation, invasion, and metastasis and in tumour immune surveillance^12,22^. Furthermore, several studies have reported that fucosylation affects T cell-mediated tumour immune surveillance through core fucosylation of T cell receptors or PD-1 on T cells^23–25^. However, given that fucosylation is difficult to quantify as a predictive marker of ICIs, it is necessary to develop a concise and minimally invasive alternative to tissue biopsies, allowing the prediction of clinical response to ICIs. Therefore, in the present study, we performed a fucosylation analysis of haptoglobin in the peripheral blood of patients with mRCC treated with anti-PD-1 monotherapy.

First, the analysis of peripheral Fuc-Hp revealed that the serum 10-7G level (Fuc-pHp) at baseline significantly affected the clinical response to nivolumab therapy, as evidenced by the lower 10-7G level in the responders (Fig. 1). Furthermore, given that the serum 10-7G level was significantly higher in patients with multiple metastases, the serum 10-7G level clearly reflects the disease progression in mRCC (Fig. 2). Previous studies have reported that fucosylation affects various cellular functions, such as cell–cell adhesion, signal transduction, and immune recognition in patients with RCC^26–28^. Therefore, Fuc-pHp appears to be a reliable biomarker for predicting the clinical efficacy of ICIs and is expected to impact the potential therapeutic effects.

Second, we found that the serum 10-7G level primarily reflected Fuc-pHp levels in the peripheral blood of patients with mRCC, as indicated in Fig. 3a,b. In contrast, a lower positive correlation was observed between serum 10-7G and Fuc-pHp levels in patients with pancreatic cancer (data not shown). Given that the 10-7G mAb partially detects Fuc-mHp, we speculate that these differences are due to the lower frequency of liver metastasis in patients with mRCC compared with those with pancreatic cancer, leading to lower production of Fuc-mHp in liver metastasis^12^. It is also known that Fuc-mHp is produced in the liver metastases due to cancer-associated inflammation and the loss of cellular polarity of hepatocytes^12^. In addition, Ito et al. also reported that Fuc-pHp is largely produced in immune cells but not in pancreatic cancer cells^12^. These results indicate that cells producing Fuc-mHp and Fuc-pHp in the tumour microenvironment may differ according to cancer type.

This study had some limitations. First, owing to the small sample size, our findings may not be generalisable to other types of cancer. Further investigations are required to validate our results in more patients. Second, we further need to establish immunohistochemistry system for evaluating Fuc-mHp-producing cells in RCC tissue microenvironment, which elucidate the composition of immune and cancer cells produce Fuc-mHp.

In conclusion, to the best of our knowledge, this is the first study to show peripheral Fuc-pHp as a predictive biomarker of clinical prognosis in patients with mRCC, enabling physicians to determine the choice of ICI treatment. For translation into clinical settings, changes in fucosylation could be a major therapeutic target in patients with mRCC.

## Methods

### Study design

In this study, we enrolled 31 patients with mRCC treated with nivolumab between June 2017 and May 2021. All patients were diagnosed with RCC through nephrectomy or needle biopsy before the initiation of nivolumab therapy. Follow-up was performed according to the standard institutional procedure for mRCC. Clinical examination, laboratory testing, and computed tomography (CT) analysis were performed every 3 months. The ORR was evaluated using the Response Evaluation Criteria in Solid Tumours (version 1.1)^29^ and defined as the proportion of patients who achieved complete response (CR) and partial response (PR). Blood samples were collected from 31 patients before and one month after the initiation of nivolumab therapy.

The study protocol was approved by the Institutional Review Board of Osaka University (approval number 668-5) and was conducted in accordance with the Declaration of Helsinki. Written informed consent was obtained from all patients.

### ELISA assays

Whole blood samples were collected in tubes containing ethylenediaminetetraacetic acid. Blood samples were centrifuged at 900 and 20,000 rpm for 10 min, and supernatants were stored at − 80 °C as plasma. A total of 1 mL of plasma was used for ELISA. *Aleuria Aurantia Lectin* (AAL)-Hp, 10-7G, and total-Hp ELISA were performed using in-house pipelines described previously^12,13^.

### Western blotting analysis and antibody

We analysed a 0.5-μL aliquot of serum on a 10% polyacrylamide gel and transferred the sample to a polyvinylidene difluoride membrane. The membrane was blocked for 1 h with 5% skim milk and was subsequently incubated with the 10-7G mAb at 4 °C overnight. As described previously, we generated the 10-7G mAb that mainly recognises Fuc-pHp to determine which cells produce Fuc-pHp^13^. After washing thrice with TBS-T, the membrane was incubated with peroxidase-conjugated anti-mouse immunoglobulin G (IgG) for 1 h. After rewashing thrice with TBS-T, bound immunoglobulins were visualised using ImmunoStar® Zeta (Wako, Osaka, Japan), according to the manufacturer's instructions. Chemiluminescent signal detection and quantification were performed using a FUSION chemiluminescence imaging system (Vilber-Lourmat, Collegien, France). The amount of Fuc-pHp in each serum sample was calculated based on the ratio of the chemiluminescence signal intensity of the sample to that of the positive control.

As the positive control, HEK293T cells were transfected with the pHp-flag expression plasmid. Twenty-four hours after transfection, cells were plated and grown on solid media containing hygromycin B (FUJIFILM-Wako, Osaka, Japan) and incubated for 2–3 weeks to obtain stable pHp-flag-expressing transfectants. The supernatant from pHp-flag-overexpressing HEK293T cells was collected, and pHp-flag was purified by affinity chromatography using ANTI-FLAG M2 Affinity Gel (Sigma-Aldrich, St. Louis, MO, USA) according to the manufacturer’s protocol. The quantity of pHp in each sample of patient sera was estimated from the ratio of the chemiluminescent signal intensities in the sample and the positive control (e.g., 5.0 ng proHp-flag) was used as described above.

As shown in raw data of western blot analysis (see Supplementary Fig. S5 online), we detected Fuc-pHp using precut membranes at the range of 40–100 kDa in order to save the 10-7G mAb.

### Statistical analysis

Statistical analysis was performed using JMP Pro 14.0.0 software (SAS Institute Inc., Cary, NC, USA). The Mann–Whitney *U* test (two-tailed) was performed to detect significant differences in 10-7G and AAL-Hp levels between responders and non-responders before and 1 month after the initiation of nivolumab therapy. Statistical significance was set at *p* < 0.05. The Kaplan–Meier method was used to calculate survival rates. The progression-free survival (PFS) was defined as the interval from the start of nivolumab therapy to death or disease progression. The log-rank test was used to compare the two groups.

### Supplementary Information

Supplementary Figures.

## Data Availability

The data generated and/or analyzed in the current study are available from the corresponding author on reasonable request.

## Abbreviations

AAL: Aleuria Aurantia Lectin
AUC: Area under the curve
ELISA: Enzyme-linked immunosorbent assay
Fuc-Hp: Fucosylated haptoglobin Hp, haptoglobin
ICI: Immune checkpoint inhibitor
PD-1: Programmed cell death protein 1
PD-L1: Programmed death ligand 1
