# Supplementary material for: Prognostic significance of serum fucosylated pro-haptoglobin in advanced renal cell carcinoma patients treated with immune checkpoint inhibitors
Source: Sci Rep. 2023 Oct 11;13:17239. doi: 10.1038/s41598-023-42739-1 (PMC10567678; doi:10.1038/s41598-023-42739-1)

**Supplementary Figure 1. Establishment of measurement system for fucosylated haptoglobin**
Schematic illustration of the fucosylated pro-haptoglobin and mature-haptoglobin evaluated using ELISA with 10-7G monoclonal antibody (mAb) and AAL antibody.

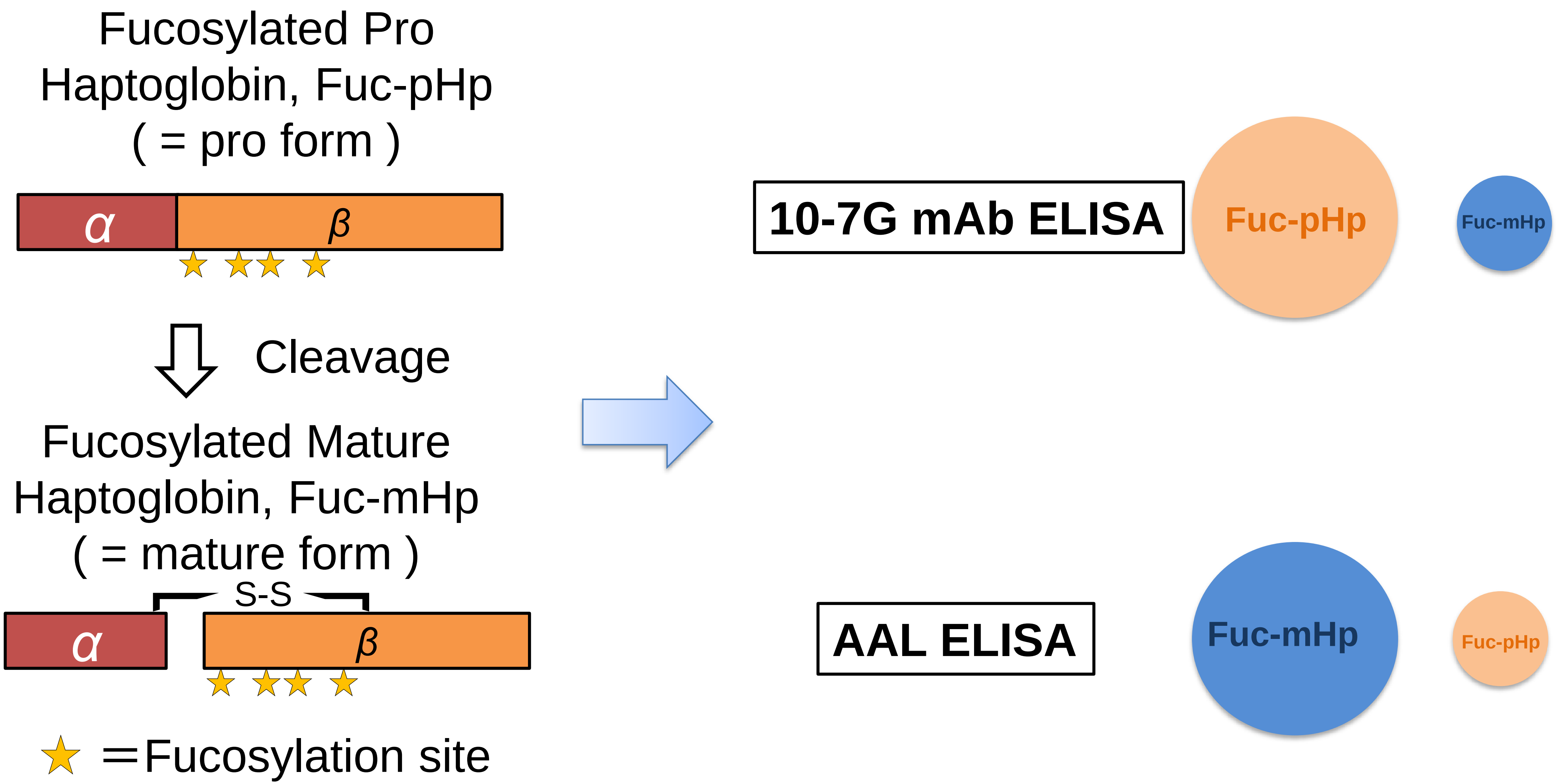

**Supplementary Figure 2. Low 10-7G level in responders who received nivolumab as second-line treatment**
Responders tended to have lower 10-7G levels in the peripheral blood before treatment initiation (vs. non-responders), even when we focused on the patients who received nivolumab as a second-line treatment. R, Responders; NR, Non-responders.

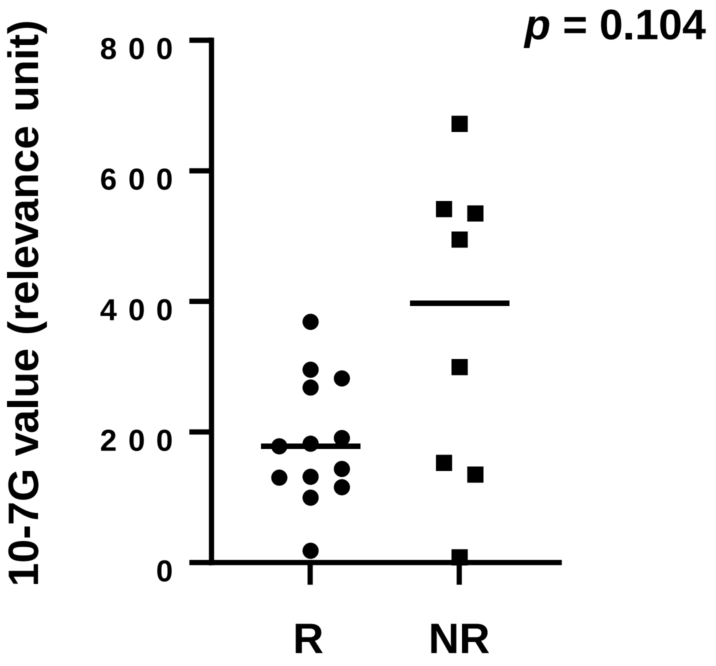

**Supplementary Figure 3. The tendency of low 10-7G level in responders at 1 month after the initiation of nivolumab monotherapy**

Responders tended to have lower 10-7G levels in the peripheral blood (vs. non-responders) at 1 month after treatment initiation. R, Responders; NR, Non-responders.

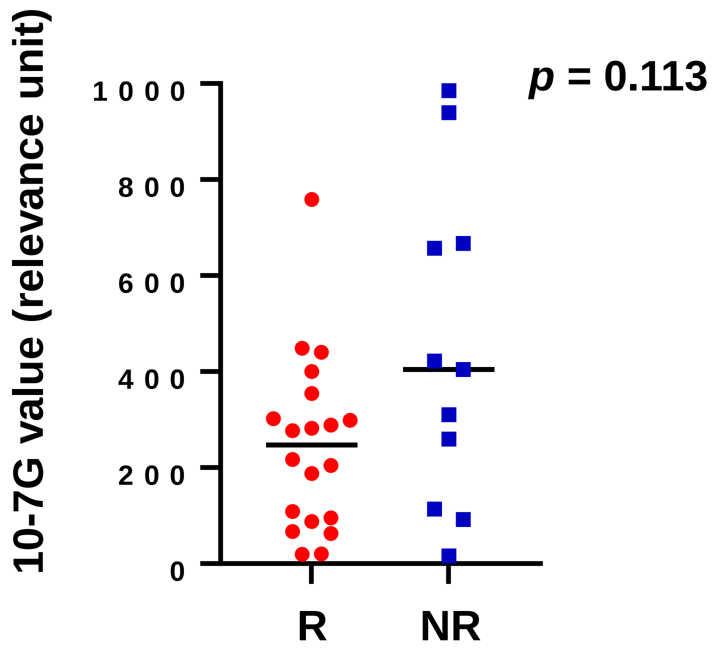

**Supplementary Figure 4. Coefficients of correlation between fucosylated pro-haptoglobin and fucosylated mature-haptoglobin**

We found no significant correlation between the 10-7G level (fucosylated pro-haptoglobin) and AAL-level (fucosylated mature-haptoglobin at baseline.

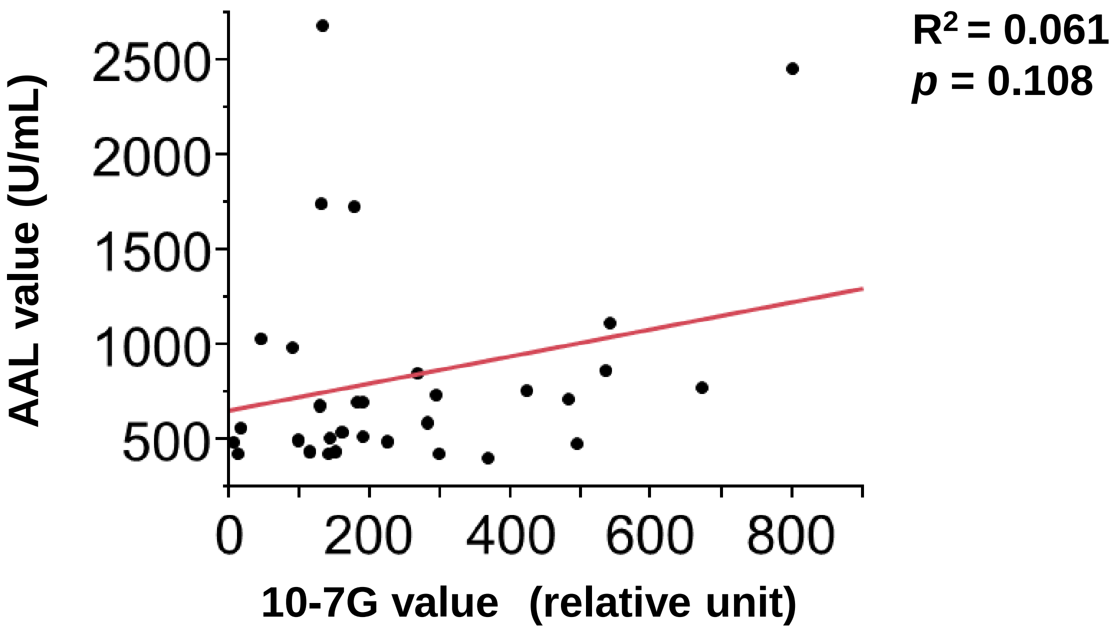

**Supplementary Figure 5. Semi-quantification of fucosylated pro-haptoglobin by western blotting (full-length membrane)**

We assessed serum fucosylated pro-haptoglobin (Fuc-pHp) levels using the 10-7G antibody using immunoblot analysis. Full-length membranes were shown in these figures. Molecular weight of Fuc-pHp is around 60 kDa.
Each line is corresponding to that in Figure. 3a.

**Line 1**

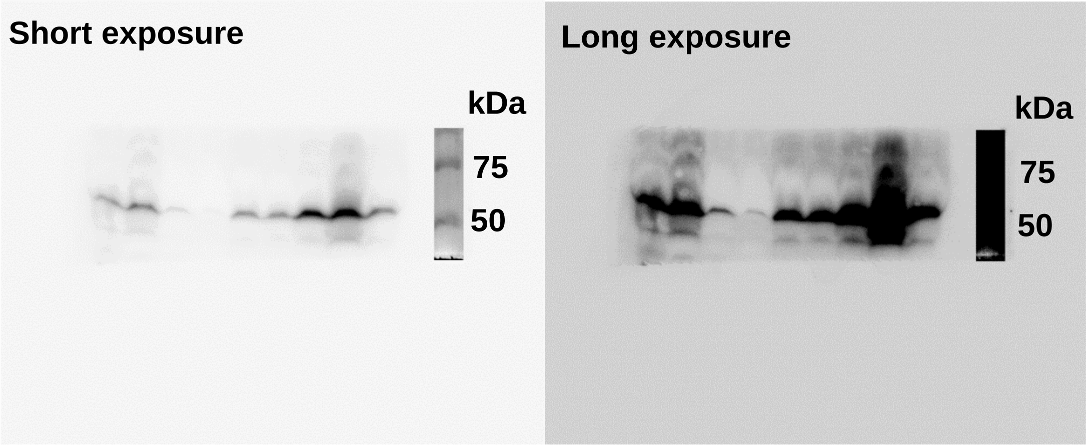


**Line 2**

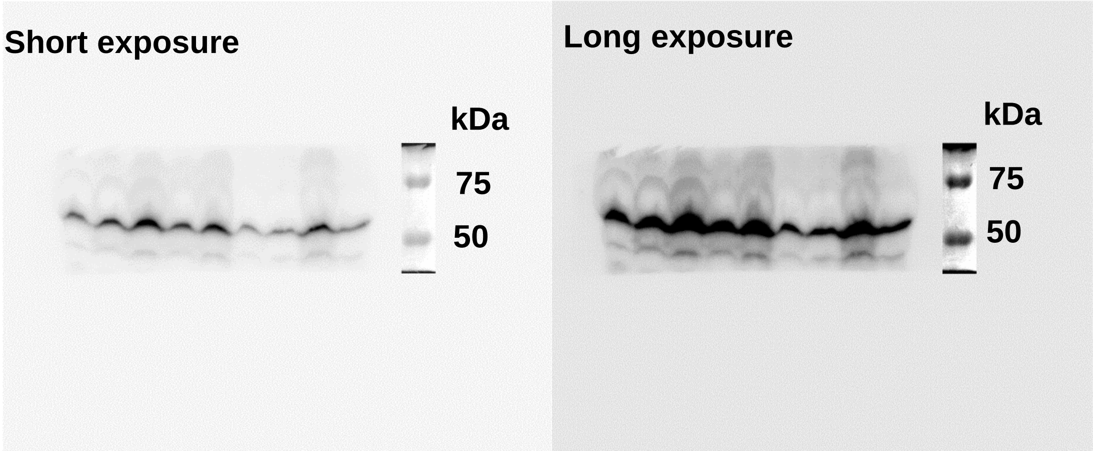


**Line 3**

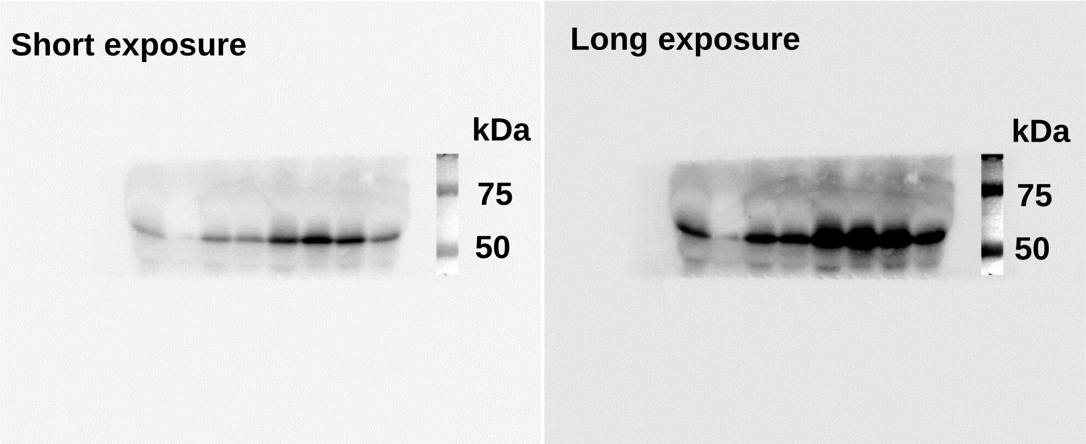


**Line 4**

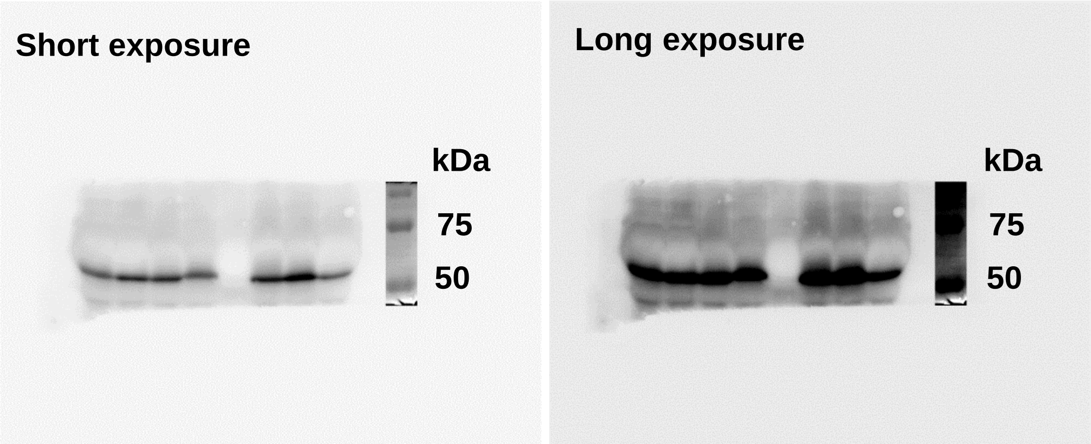

Supplement: Supplementary file 1 — Supplementary Figures. [file 41598_2023_42739_MOESM1_ESM.pdf]
